# Persistence of *Legionella* in Routinely Disinfected Heater-Cooler Units and Heater Units assessed by Propidium Monoazide qPCR

**DOI:** 10.3390/pathogens9110978

**Published:** 2020-11-23

**Authors:** Savina Ditommaso, Monica Giacomuzzi, Gabriele Memoli, Jacopo Garlasco, Carla M. Zotti

**Affiliations:** Department of Public Health and Pediatrics, University of Turin, 10126 Torino, Italy; monica.giacomuzzi@unito.it (M.G.); gabriele.memoli@unito.it (G.M.); jacopo.garlasco@unito.it (J.G.); carla.zotti@unito.it (C.M.Z.)

**Keywords:** *Legionella*, heater–cooler unit, propidium monoazide qPCR, NTM

## Abstract

Background: Evidence to date indicates that heater–cooler units (HCUs) and heater units (HUs) can generate potentially infectious aerosols containing a range of opportunistic pathogens such as *Mycobacterium chimaera*, other non-tuberculous mycobacterial (NTM) species, *Pseudomonas aeruginosa* and *Legionella* spp. Our purpose was to determine the extent of *Legionella* contamination and total viable count (TVC) in HCUs and HUs and to analyze the relationship by water system design of devices of two different brands (LivaNova vs. Maquet). Methods: *Legionella* spp. were detected and quantified by our optimized PMA-qPCR protocol; TVCs were assessed according to ISO protocol 6222. Analyses were performed in the first sampling round and after six months of surveillance. Results: Overall, *Legionella* spp. was detected in 65.7% of devices. In the second sampling round, *Legionella* positivity rates were significantly lower in water samples from the Maquet devices compared to the LivaNova ones (27.3% vs. 61.5%). LivaNova HCUs also yielded more *Legionella*, and aquatic bacteria counts than Maquet in both first and second-round samples. Conclusions: We recommend that all surgical patients and staff exposed to aerosols from thermoregulatory devices should be followed up for *Legionella* infection and that microbiological surveillance on such devices should be conducted regularly as precautionary principle.

## 1. Introduction

*Legionellae* are ubiquitously present in water environments, either natural or man-made. Even though these bacteria are usually found at low concentrations in natural aquatic environments, mounting evidence has shown how artificial water environments with particular physical and chemical conditions can favor the growth of these pathogens [1]. Documented sources include cooling towers [2,3,4], hospital water systems [5], swimming pools [6,7], domestic water systems and showers [8,9], ice-making machines [10,11], whirlpool spas [12,13], hospital pool water for water birthing [14,15], hot springs [16,17], fountains [18], dental units [19], soil [20], automobile windshield washer fluid [21] and wastewater treatment plants [22].

In many nosocomial *Legionella* outbreaks [5,23] water distribution systems are the most frequent source of infection. The key to legionellosis prevention are proper maintenance of water systems in which *Legionella* spp. grows (disinfection, water system supply maintenance), staff training and the implementation of a clinical surveillance [24].

Evidence to date indicates that heater–cooler units (HCUs) used during cardiopulmonary bypass surgeries can generate potentially infectious aerosols containing a range of opportunistic pathogens such as *Mycobacterium chimaera* [25], other non-tuberculous mycobacterial (NTM) species [26,27], *Pseudomonas aeruginosa*, *Stenotrophomonas maltophilia* and fungi [28,29]. In the US and Europe, non-tuberculous mycobacteria (NTM) infections have been identified in patients undergoing cardiothoracic surgeries [28,30].

Heater units (HUs) are used in extracorporeal membrane oxygenation (ECMO), to provide prolonged cardiopulmonary support in acute respiratory distress syndrome. To date, there have been three reports of HU contamination with *Mycobacterium chimaera* world-wide [31,32,33] yet any link between HU contamination and patient infection with *Mycobacterium chimaera* is still to be determined.

The isolation of *Legionella* spp. from several HCUs [28,34,35] has raised concern that also these pathogens may be transmitted to the surgical staff through aerosolization of the water contained in these devices. While it is unlikely that respiratory exposure to *Legionella* from HCUs occurs in patients undergoing cardiothoracic surgery due to the closed-circuit ventilation of these units, there is also a theoretical risk of exposure to *Legionella*-containing aerosols for same-unit patients that are not being kept on closed circuit ventilation.

To date, however, there is lack of consistent evidence supporting a correlation between airborne *Legionella* transmission in operating theatres and HCU or HU usage. According to Public Health England surveillance data (1 January 2007 to 1 November 2016), no cases of Legionnaires’ disease were identified in healthcare workers expected to have been exposed to *Legionella* in cardiac surgery setting, and no *Legionella*-derived endocarditis cases were reported in cardiothoracic surgery patients [36]. In the U.S., at the University of Washington Medical Center, Seattle, two out of four *Legionella*-infected patients had previously undergone surgery involving the use of CardioQuip HCUs positive for *Legionella* spp. [34]. Of note, the manufacturer of these devices, had recently been implicated in three NTM (*Mycobacterium abscessus*) patient infections at a single facility involving the use of the MCH-1000 devices CardioQuip (Bryan, TX, USA). The Food and Drug Administration (FDA) has alerted healthcare providers to the risk of infection when using CardioQuip’s heater–cooler device during cardiothoracic surgery [37].

As the aforementioned reports leave open the possibility that *Legionella* transmission may occur even in the absence of a direct contact of the patient/care provider with the HCU and HU water, it has been proposed that contaminated devices’ water may leak onto other parts of the device, thereby favoring bacterial spread in the operating theatre through the aerosol [38].

Here, we report the results of the first molecular surveillance of *Legionella* contamination of water from HCUs and HUs devices using a propidium monoazide quantitative polymerase chain reaction (PMA-qPCR) method. Our study purpose was to determine the extent of *Legionella* contamination in HCUs and HUs devices while assessing the long-term efficacy of the recommended decontamination protocols against this pathogen. We also measured the total viable counts (TVCs) of aerobic heterotrophic bacteria at 36 °C and 22 °C to evaluate the relationship between *Legionella* contamination and the microbial quality of the water samples.

## 2. Materials and Methods

### 2.1. Devices

Thermoregulatory devices, heater units (HUs) or heater–cooler units (HCUs) are engaged to adjust the blood temperature within the extracorporeal circuits in acute respiratory distress syndrome, in cardiopulmonary bypass and during cardiac surgery. The devices include tanks (capacity around 24 L) that provide temperature-controlled water to external heat exchangers or to warming/cooling blankets through closed water circuits. The devices heat the water up to 41 °C, an optimal condition for multiplication of bacteria such as *Mycobacteria* spp. and *Legionella* spp. Pumps that move water into the patient’s circuit can create aerosol that is pushed out of the devices by cooling fans, thereby favoring the aerodispersion of bacteria in the operating room. This occurs when the water tanks of the devices are not closed with a sealing lid.

**Figure 1:**
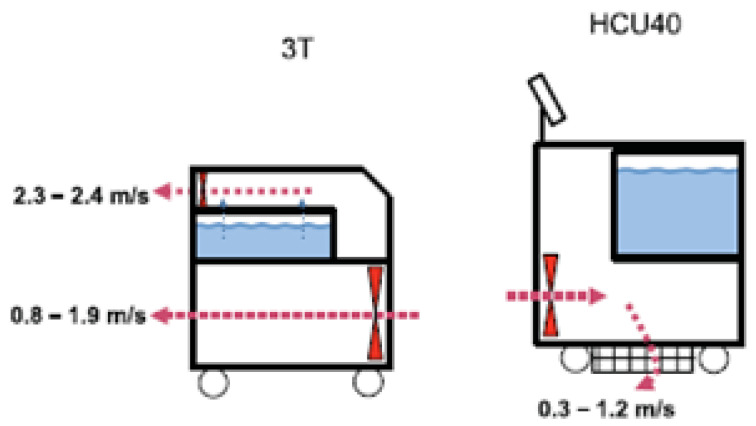
Detailed description of heater–cooler units (HCU) devices: common features and differences (ventilators in red, water tank in blue, red dotted arrows representing airflow direction, blue dotted arrows representing potential direction of aerosol leakage) (source: Kuhel et al. 2018) [39].

The HCUs from different brands can produce and release aerosol with different modality (Figure 1) as shown in previous studies [28,39] in which relevant differences in HCU40 design compared to 3T were identified: air flow direction, location of cooling ventilators, continuous cooling of the water tank at 4 °C and an electronic alarm that activates when disinfection is required.

**Figure 2:**
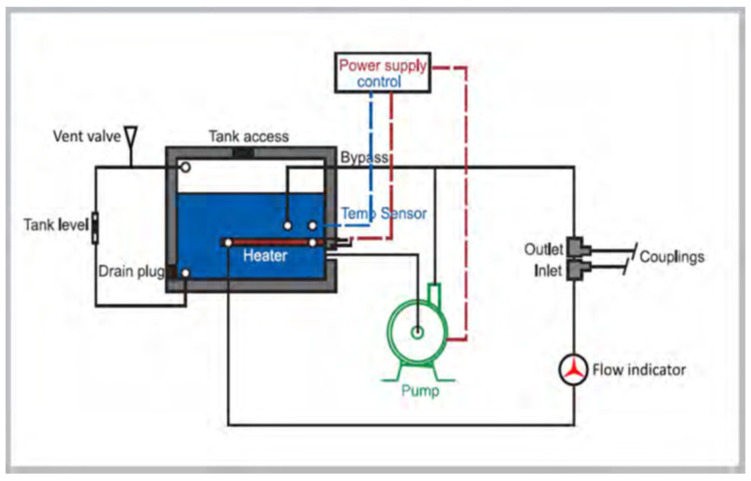
Detailed description of HU35 devices. (Provided by Getinge).

Regarding HU35, the design itself may ensure patient safety during ECMO (Figure 2) since ventilators used to cool the sealed water tank (capacity around 1.5 L) are located directly under the tank.

The disinfection of devices is performed according to the manufacturers’ updated procedures: (a) for the HCU Stockert 3T, disinfection with peracetic acid (3.3% of Puristeril 340; Fresenius Medical Care, Bad Homburg, Germany) or with sodium hypochlorite (1.3% of Clorox regular bleach, Clorox Co., Oakland, CA, USA) every 14 days, along with weekly changes of water in the presence of 100 ppm of hydrogen peroxide is recommended [40]; (b) for the HCU40, the disinfection protocol includes a weekly treatment with 2% chloramine-T [41]; (c) for the HU35, the disinfection protocol includes a treatment with 2% chloramine-T and to be performed weekly and after each single use.

The thermoregulatory devices tanks are filled, according to the manufacturer’s recommendations, with filtered water with a terminal filter of pore size of 0.2 µm.

We analyzed water samples taken from 35 thermoregulatory devices, of which 13 were HCU Stockert 3T manufactured by LivaNova PLC (Sorin Group Deutschland GmbH, Norderstedt, Germany), 8 were HCU40 and 14 were HU35 both manufactured by Maquet Getinge Group (Rastatt, Germany).

### 2.2. Water Sampling

Follow-up investigation was carried out in nine cardiac surgery facilities and one pediatric cardiac surgery suite, all located in the Piedmont region (northwestern Italy). Thirty-five devices (21 HCU and 14 HU) were analyzed in the context of periodic analyses in our laboratory from 2017 to 2020. For each HCU, one liter of water sample was collected from both circuits (i.e., the patient circuit and the cardioplegia circuit) before disinfection, whereas for HU devices a water sample of 0.5 liter was collected. Each sample was stored in a sterile plastic bottle containing sodium thiosulfate (10% *w*/*v*) to neutralize chlorinated water which may change the microbe numbers during storage from the time of sampling to the time of analysis.

In this study, we assessed bacterial contamination in water samples obtained from HCUs and HU devices at the first sampling round (before disinfection) and compared it with that in samples obtained after six months of surveillance, hereafter defined as second sampling round. During this surveillance period, the surgical personnel carried out monthly disinfection cycles of the devices according to the manufacturer’s instructions.

### 2.3. Sample Preparation, DNA Extraction and PMA Treatment

The samples were processed for *Legionella* detection using our optimized PMA-qPCR protocol, as previously described [42]. Briefly, water sample was filtered through a 0.45 μm polycarbonate filter (Millipore, Billerica, MA, USA), overlaid with 500 μL of propidium monoazide (PMA) (50 μM) in 90 mm Petri dishes and incubated in the dark for 10 min followed by a 10 min exposure to a 500 W light on ice at a distance of 20 cm from the light source. After irradiation, the filter was added to the lysis buffer for DNA extraction according to the manufacturer’s instructions (Aquadien™, Bio-Rad, Marnes-la-Coquette, France). Extracted genomic DNA was then analyzed by qPCR to detect the presence of amplifiable sequences.

### 2.4. Detection and Quantification of Legionella by qPCR

The analyses were performed by “iQ-Check™ Quanti *Legionella* spp.”, according to the manufacturer’s instructions (Bio-Rad, Marnes-la-Coquette, France). The iQ-Check™ Quanti *Legionella* spp. kit amplifies and quantifies a fragment of approximately 100 bp from the 5S rRNA gene of *Legionella* spp. using molecular beacon probes. The qPCR data were then analyzed by Bio-Rad CFX Manager IDE (Bio-Rad, Hercules, CA, USA).

### 2.5. Total Viable Count (TVC)

One milliliter of undiluted samples and 1 mL of 1:10 diluted samples, in Page’s saline solution, were tested on yeast extract agar using the pour plate method according to the UNI EN ISO protocol 6222 [43]. The number of colony-forming units (CFUs) per sample was calculated after 7 days of incubation at 22 °C or after 5 days of incubation at 36 °C, according to the US standard method [44]. Results were reported as CFU/mL.

### 2.6. Statistical Analysis

The statistical software R (version 4.0.2) [45] was used to perform all calculations and draw all plots. The proportions were compared using Fisher’s exact test. The Mann–Whitney U test was adopted to evaluate between-sample differences (LivaNova vs. Maquet) in *Legionella* quantification (genomic units per liter, GU/L) and TVC data. The correlation between respective *Legionella* counts and TVCs was evaluated using Kendall’s *tau* correlation coefficient. Finally, correlation differences between the two sampling rounds (first vs. second) were investigated by Dunn and Clark’s Z test for correlation coefficients in dependent samples [46], as implemented by the R package “cocor” [47], after converting each *tau* coefficient into the corresponding parametric coefficient *r*, as described by Walker [48]. For all tests, the level of significance was set at α = 0.05.

## 3. Results

*Legionella* spp. was detected in 65.7% of devices, with a slightly higher percentage of *Legionella*-positive samples observed in LivaNova vs. Maquet devices (76.9% vs. 59.1%).

When we analyzed a second water sample taken from the same devices after six months of monthly disinfection, we found on average a lower total number of contaminated devices (40.0%) compared to the first sampling round, albeit this difference only attained borderline significance (*p* = 0.0547).

We next analyzed the relationship between the different water system design of devices’ brand and the contamination rate. Interestingly, the percentage of contaminated machines in the second sampling round varied consistently (*p* = 0.0666) only among Maquet devices. Fully detailed results by device and brand are summarized in Table 1.

The comparison between specific thermoregulatory devices (Stockert 3T, HCU40 and HU35) did not yield statistically significant difference both in the first and in the second sampling round (data not shown).

**Figure 3:**
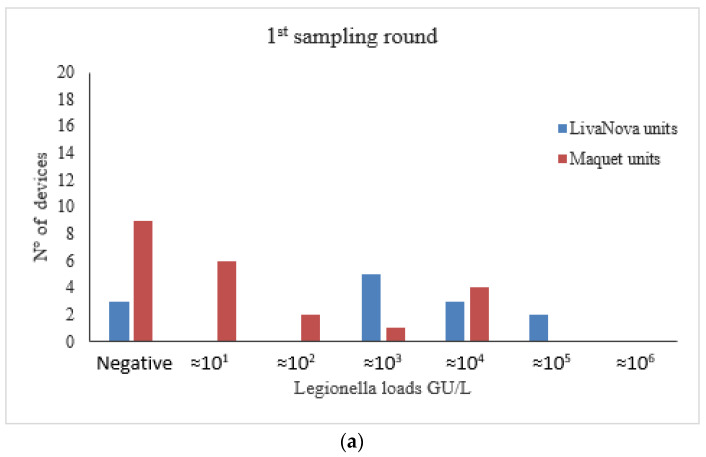

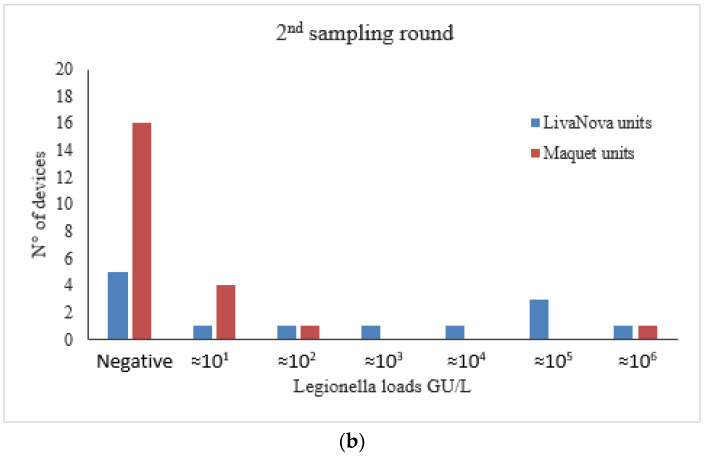
*Legionella* spp. concentration in thermoregulatory devices water samples collected at the first sampling round. (**a**) and after six months of monthly disinfection at the second sampling round (**b**).

*Legionella* load ranged from 1.2 × 10^1^ to 5.9 × 10^6^ GU/L. Data frequency distributions are shown in Figure 3a,b.

Samples collected from LivaNova maintain higher microbial loads despite the disinfection cycles, compared to Maquet device. The statistical analysis showed a significant difference compared to the concentrations of *Legionella* found in the Maquet ones (Figure 3b, Table 2).

Counts relative to the first and second sampling round revealed that the HCUs manufactured by LivaNova had a significantly higher counts of both aquatic bacteria and *Legionella* compared to Maquet devices (Table 2).

As the TVC analysis showed a clear correlation between the results obtained at 22 °C and 36 °C in both the first (τ = 0.9592; *p* < 0.0001) and second round (τ = 0.8809; *p* < 0.0001), we opted to report and discuss hereafter only the TVC results relative to the 22 °C condition.

**Figure 4:**
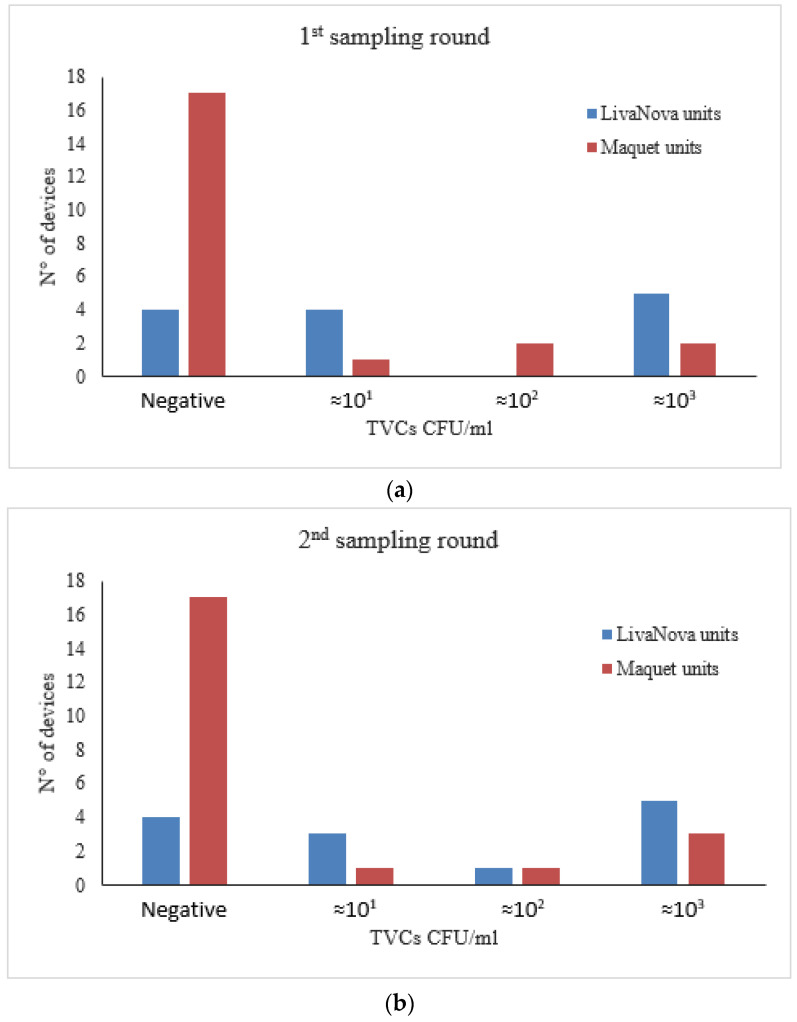
TVC concentration in thermoregulatory devices water samples collected at the first sampling round. (**a**) and after six months of monthly disinfection at the second sampling round (**b**).

Overall, aquatic bacteria were detected in 14 of the 35 water samples (40.0%) from both the first and second sampling round (Table 3). Importantly, the devices manufactured by LivaNova yielded a significantly higher number of positive samples (≥1 CFU/mL) than those manufactured by Maquet (*p* = 0.012), again with identical results for each sampling round (Table 3, Figure 4a,b).

**Figure 5:**
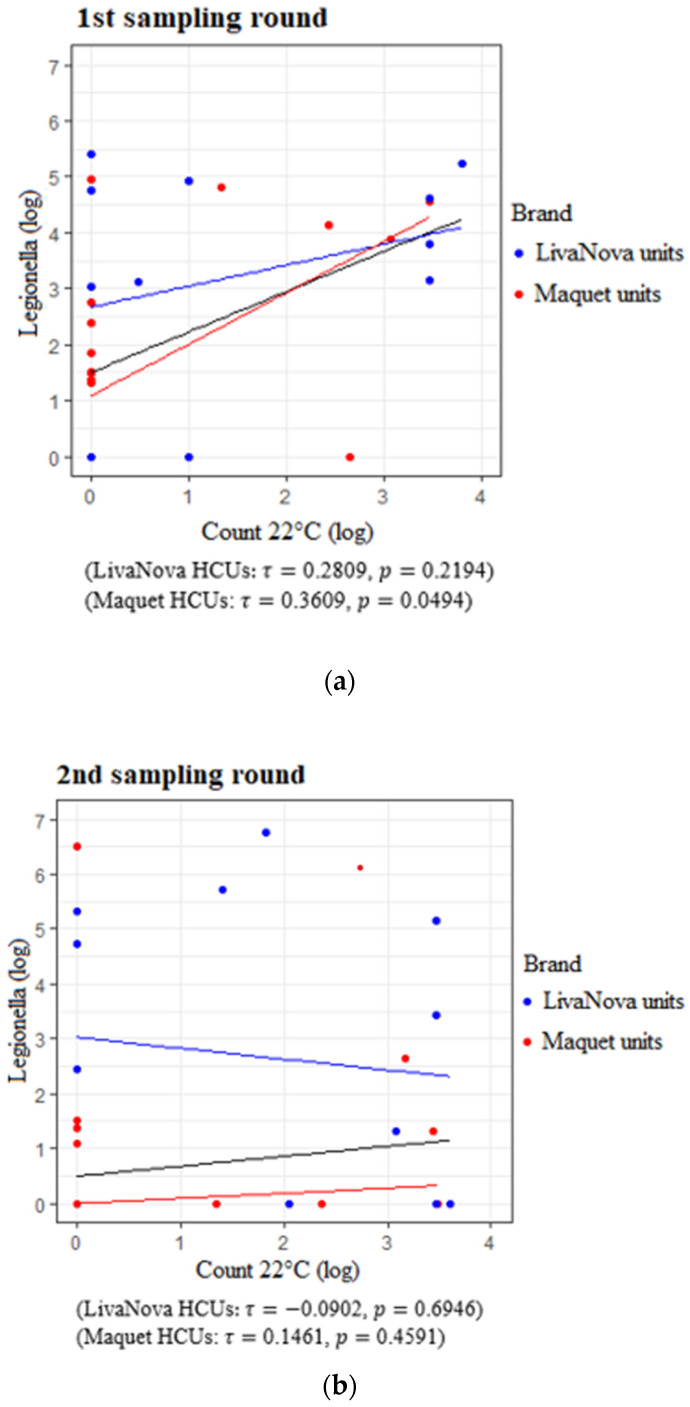
(**a**) Correlation between Legionella counts and TVC at 22 °C in the first sampling round. (**b**) Correlation between *Legionella* counts and TVC at 22 °C in the second sampling round.

*Legionella* counts and TVC in water samples from the 1st and 2nd sampling round at 22 °C appeared to be positively correlated, with a moderate association reaching statistical significance (*τ* = 0.3984, *p* = 0.0038) (Figure 5a). This correlation was more evident among Maquet devices (*τ* = 0.3609, *p* = 0.0494), whereas it was weaker and not significant in LivaNova devices (*τ* = 0.2809, *p* = 0.2194). In contrast, the same analysis failed to find any correlation between TVC at 22 °C and *Legionella* counts in samples drawn during the second round, either considering all samples (*τ* = 0.1663, *p* = 0.2489) or one of the two brands (LivaNova: *τ* = −0.0902, *p* = 0.6946; Maquet: *τ* = 0.1461, *p* = 0.4591) (Figure 5b).

Finally, Dunn and Clark’s Z test for dependent samples detected a difference between the respective correlation coefficients of the two sampling rounds, although not statistically significant (*p* = 0.0831) probably due to the limited sample size (*n* = 35).

## 4. Discussion

High microbial load in HCU or HU water, possibly containing pathogenic bacteria such as NTM and *Legionella* spp., poses a potential risk of infection for patients and personnel in operating theatres. This contamination can be mainly ascribed to the presence of waterborne bacteria: the microorganisms in HCU and HU biofilms primarily originate from tap water when tap water is added to the water tanks of thermoregulatory devices without filtration [49,50], a procedure strongly discouraged by the manufacturers. However, the risk of aerosol emission is related to the design of water system devices and to the internal ventilation systems [25,39,51]. In fact, the risk of infection arising from the use of HU35 is foresee-ably low, due to the air-tight and closed system design device (without the external cooling fan) and the minimally invasive nature of ECMO [52].

FDA began receiving reports of non-tuberculous mycobacteria (NTM) infections linked to the devices in 2010, leading to a 2015 warning letter to LivaNova [53] and subsequent design changes intended to cut the risk. Regulatory communications on the topic have centered on LivaNova, but FDA has also received reports of contamination linked to devices from Cincinnati Sub-Zero, Maquet and Terumo [54].

In order to reduce the risk of aerosol transmission, LivaNova has recently upgraded its HCU 3T manufactured before 2016 with a new internal sealing. In addition, it has equipped them with a vacuum pump aspirating liquids into the central suction system of the hospital, which reduces—albeit not fully eliminating—the exhaust emissions from the rear of the machine [53]. On 25 February 2020 LivaNova received FDA clearance for 3T heater–cooler device modification [55]. Although regular and effective decontamination and microbiological surveillance is crucial to mitigate the risk of infection due to *M. chimaera* [32,56,57] and other opportunistic pathogens, such as *Legionella*, the theoretical risk of aerosolization remains high when these devices are decontaminated [58,59] or emptied, or when their circuits are damaged.

This study is the first to quantify *Legionella* in HCUs and HU35 devices using the PMA-qPCR method. Previous reports assessed the extent of *Legionella* contamination in thermoregulatory devices using either bacterial culture [28] or PCR [35], with the latter method being used to examine three devices only. In our study, besides analyzing a much larger number of devices from numerous regional hospitals, we were able to inhibit PCR amplification of DNA from dead cells by including PMA treatment in our protocol, which allowed us to obtain a *Legionella* recovery rate of 65.2%. Thus, based on our data, we strongly recommend using the PMA-qPCR method to achieve optimal detection and quantification of *Legionella* in artificial aquatic environments.

Between-brand comparison (LivaNova vs. Maquet) of *Legionella* positivity rates showed that the proportion varied consistently in the second sampling round (Table 1), with a significantly lower percentage of *Legionella*-positive water samples from the Maquet devices compared to those by LivaNova (27.3% vs. 61.5%). The differences in *Legionella* positivity rates between thermoregulatory water systems (Stockert 3T, HCU 40 and HU 35) will be investigated on further water samples collected during the regional surveillance and analyzed by our laboratory (Regional Reference Laboratory).

The HCUs manufactured by LivaNova also yielded higher *Legionella* and aquatic bacteria counts than those manufactured by Maquet in both first- and second-round samples, a difference that was statistically significant (Figure 3 and Figure 4; Table 2). Moreover, we found a positive correlation between *Legionella* and TVCs in first-round samples, in particular among Maquet devices (Figure 5a). These results confirm the findings of a previous study on dental units showing that the concentration of heterotrophic bacteria is associated with *Legionella* when the detection of this pathogen is carried out by PCR [19]. The lack of correlation between *Legionella* and TVCs in the second-round samples (Figure 5b) is probably due to a difference in the long-term effectiveness of the disinfection program against aquatic flora [49] compared to that used for *Legionella*.

The results obtained in this study showed that the decontamination protocol recommended by LivaNova (3.3% Puristeril 340), and systematically implemented by cardiac surgery teams, failed to significantly decrease the microbial load (including *Legionella*) during the six months of this observation. In our previous analysis [36], we demonstrated that the disinfection procedures were effective in reducing TVCs of bacteria in both LivaNova and Maquet devices, with the exception of NTM species. However, in that instance, we had analyzed water samples obtained shortly after disinfection and compared them with the pre-disinfection condition. Instead, samples analyzed in the present study were collected before disinfection and after six months of monthly disinfection, with matched comparisons showing that these devices can be recolonized during this time period.

These results are in line with what has been previously observed in dental unit waterlines and flexible endoscopes [19,60,61,62,63,64], where waterborne bacteria persisting in waterline effluent water, despite previous disinfection, can efficiently recolonize the water circuits of these devices. Given the well-established tolerance of biofilm to disinfectants, it is tempting to speculate that *Legionella* recolonization may be due to biofilm formation and persistence in these devices. Indeed, following continuous exposure to antimicrobial agents, biofilms have been shown to favor the selection of resistant bacteria that can repopulate the water environment thought to have been decontaminated.

Probably the difference in microbial load observed between LivaNova and Maquet devices is to be ascribed to the use of peracetic acid (Puristeril) for disinfecting LivaNova devices. Some published studies on fixative properties of disinfectants have, in fact, confirmed that organic polymers forming the biofilm matrix might be coagulated by the acidic pH of peracetic acid, thereby creating a greater barrier for the disinfectant to reach bacterial cells [65,66,67]. This phenomenon could be due to a lower efficacy of the disinfectant solution in removing the components of mature biofilms and/or to a modification of the biofilm structure after repeated treatments: the latter would make it less sensitive to the action of the peracetic acid formulation. In our experience, 3.3%-diluted Puristeril maintains a pH of ≈3.5, which may have contributed to biofilm consolidation.

## 5. Conclusions

In conclusion, here we report significant *Legionella* spp. contamination in water samples from previously disinfected thermoregulatory devices. This study confirms that the manufacturer’s disinfection procedures fail to be effective in the long term against *Legionella*, as well as other pathogens, particularly for LivaNova Stockert 3T HCUs, which seem to provide a greater chance for microbial recolonization and biofilm consolidation during the intervals between disinfections.

The lack of Legionnaires’ disease case in the surgical setting may lead one to believe that the risk of *Legionella* exposure for surgical staff and patients is actually very low, though it must be remembered that Legionnaires’ disease is likely to be underdiagnosed, and the true incidence may be underestimated. Therefore, the results of the present study seem to suggest that microbiological surveillance on HCUs or HU devices should be conducted regularly as a precautionary principle. Further analyses are needed to confirm the impact of the device’s design on the possibility of *Legionella* transmission so as to determine the extent to which surgical patients and staff exposed to aerosols from these devices should be followed up for legionella infection.

## Figures and Tables

**Table 1:** Frequency of *Legionella*-positive devices according to brand. Fisher’s exact test was used for between-round and between-brand (LivaNova vs. Maquet) positivity rate comparisons.

	Total	LivaNova Units	Maquet Units	Comparison between Brand *p*-Value
Number of units	35	13	22	
1st round *Legionella*-positive (%)	23 (65.7)	10 (76.9)	13 (59.1) *	0.4630
2nd round *Legionella*-positive (%)	14 (40.0)	8 (61.5)	6 (27.3) **	0.0751
comparison between round (*p*-value)	0.0547	0.6728	0.0666	

* (5 HCU 40 and 8 HU35) ** (3 HCU 40 and 3 HU35).

**Table 2:** *Legionella* counts and TVC in water samples from the 1st and 2nd sampling round. Mann–Whitney–Wilcoxon U test was used for between-brand (LivaNova vs. Maquet) count comparisons.

	Count Type	All Units Median (Q1–Q3)	LivaNova Units Median (Q1–Q3)	Maquet Units Median (Q1–Q3)	Comparison between Brand *p*-Value
1st round	*Legionella*	72 (0–10,615)	1418 (1113–57,824)	28 (0–497)	*p* = 0.037
	TVC (22 °C)	0 (0–149)	10 (0–3000)	0 (0–0)	*p* = 0.010
2nd round	*Legionella*	0 (0–156)	279 (0–138,912)	0 (0–9)	*p* = 0.020
	TVC (22 °C)	0 (0–170)	67 (0–3000)	0 (0–0)	*p* = 0.011

**Table 3:** Frequency of total viable count (TVC)-positive devices according to brand. Fisher’s exact test was used for between-brand (LivaNova vs. Maquet) positivity rate comparisons.

	Total	LivaNova Units	Maquet Units	Comparison between Brand *p*-Value
Number of units	35	13	22	
1st round TVC-positive (%)	14 (40.0)	9 (69.2%)	5 (22.7%) *	0.012
2nd round TVC-positive (%)	14 (40.0)	9 (69.2%)	5 (22.7%) **	0.012

* (4 HCU40 and 1 HU35) ** (4 HCU40 and 1 HU35).
